# Bimodal intensity-modulated radiotherapy in combination with carbon ion therapy (C12) of mucosal melanomas - data of the last decade from Heidelberg University Hospital

**DOI:** 10.3389/fonc.2024.1437412

**Published:** 2024-11-22

**Authors:** Lukas Bauer, Angela Paul, Sebastian Regnery, Maximilian Y. Deng, Malte Ellerbrock, Thomas Mielke, Semi B. Harrabi, Katharina Seidensaal, Thomas Held, Klaus Herfarth, Jürgen Debus, Jessica C. Hassel, Kristin Uzun-Lang

**Affiliations:** ^1^ Department of Radiation Oncology, Heidelberg University Hospital, Heidelberg, Germany; ^2^ Heidelberg Institute of Radiation Oncology (HIRO), Heidelberg, Germany; ^3^ National Center for Tumor Diseases (NCT), Heidelberg, Germany; ^4^ Heidelberg Ion-Beam Therapy Center (HIT), Department of Radiation Oncology, Heidelberg University Hospital, Heidelberg, Germany; ^5^ Clinical Cooperation Unit Radiation Oncology, German Cancer Research Center (DKFZ), Heidelberg, Germany; ^6^ Section of DermatoOncology, Department of Dermatology and National Center for Tumor Diseases, University Hospital Heidelberg, Heidelberg, Germany

**Keywords:** mucosal melanoma, carbon ion therapy, bimodal radiotherapy, radiotherapy, particle therapy

## Abstract

**Introduction:**

Due to the rarity of mucosal melanomas, few recent studies can be found investigating the success and side effects of therapy for this entity with large numbers of patients. In this retrospective analysis, the efficacy and toxicity of combined intensity-modulated radiotherapy (IMRT) and carbon ion therapy (C12) of mucosal melanomas were analyzed to contribute to a better understanding of this rare disease.

**Methods:**

Twenty-two patients were included from 2013 to 2022 in the Department of Radiation Oncology at Heidelberg University Hospital. 19 patients received bimodal radiotherapy consisting of radiotherapy by IMRT and carbon ion therapy (C12). 3 patients received photon only IMRT. In addition to Overall Survival (OS), local control rate (LCR), locoregional control rate (LRCR) and progression-free survival (PFS), early and late toxicity of treatment was analyzed. Bimodal radiotherapy consisted of IMRT of the primary tumor region and cervical lymph nodes in a single dose of 2 Gy up to the dose of 50.0 Gy in the basic schedule after application of a C12 boost of the primary tumor region up to 24 Gy (RBE) in a single dose of 3 Gy (RBE) up to the total dose of 74.0 Gy (RBE) in 5-6 fractions/week. Photon only radiotherapy comprised IMRT up to total doses of 66-70,4 Gy in 5 fractions/week.

**Results:**

After 2 years, overall survival, progression-free survival, local control and locoregional control were 46%, 41%, 77% and 77%, respectively. 4 out of 5 patients with local relapse showed in-field recurrence inside the C12 boost volume. The primary tumor in these patients was always located in the main nasal cavity and/or paranasal sinus. Leading acute toxicity was grade 2 mucositis (12 patients, 55%) followed by grade 1 radiation dermatitis (10 patients, 45%). The cumulative incidence of late grade 3 toxicities was 15%.

**Discussion:**

The combination of IMRT with carbon ion therapy in the treatment of mucosal melanoma provides promising local control rates with mild acute toxicity despite unfavorable patient preselection. The unfavorable overall survival as well as progression-free survival rates indicate that concomitant systemic therapies should be the subject of future research.

## 1 Introduction

Mucosal melanoma (MM) of the paranasal sinus is an exceedingly rare condition, with an annual incidence of less than 1 case per 100,000 people (1). Unfortunately, most cases are not detected until they have reached an advanced stage, resulting in a generally bleak prognosis, with 5-year survival rates ranging from 20% to 36% (2, 3).

Poor treatment outcomes after the established treatment with aggressive surgery followed by adjuvant radiotherapy (RT) urged the need for systemic therapy in mucosal melanoma patients. With the emerging field of immunotherapies, hopes were up to find efficient and tolerable systemic approaches e.g. neoadjuvant checkpoint inhibitor application. However, neoadjuvant systemic therapy for MM is not well-studied. As one of the few studies, Ho et al. showed an 3-year-OS of 55% after neoadjuvant anti-PD1 +/- anti-CTLA4 (4). Nevertheless, further research and exploration are warranted to substantiate these findings fully.

Adjuvant radiotherapy in patients with MM faces considerable challenges, particularly when dealing with advanced tumor stages. One notable issue in achieving effective local control for these tumors is the limitation on dose escalation due to the presence of nearby critical structures. Particle therapy, specifically carbon ions (C12), offers unique advantages in this context. Carbon ions combine two critical physical properties: they create steep dose gradients that allow for dose escalation within the paranasal sinuses without a corresponding increase in side effects, and they possess a higher linear energy transfer (LET), resulting in increased biological effectiveness in targeting cancer cells (5–7).

As a result of these advantages, there has been a surge of interest in using particle therapy, particularly C12 therapy, to treat mucosal melanoma in the paranasal sinuses. More recently, Takayasu et al. reported a 3-year-OS of 49.2% after Carbon-ion radiotherapy with concurrent dacarbazine, nimustine, and vincristine therapy in 21 clinically localized MM patients between 2012 and 2019 (8). Earlier investigations into carbon-ion radiotherapy for head and neck mucosal melanoma have demonstrated local control rates up to 84% (9).

Contributing to a better understanding of this rare disease, we did a retrospective analysis including the efficacy and toxicity of radiotherapy, especially combined intensity-modulated radiotherapy and carbon ion therapy (C12), of mucosal melanomas at Heidelberg University Hospital.

## 2 Materials and methods

### 2.1 Screening

A total of 53 patients with diagnosed mucosal melanoma (MM) were screened from the NCT database. The retrospective analysis comprised all patients that received radiotherapy at our clinic. Patients with previous radiotherapy of the MM or discontinuance of radiotherapy were excluded. Between 2013 and 2022, 22 patients were treated with radiotherapy at our clinic.

### 2.2 Patient characteristics

In total, 22 patients (32% males) with a median age of 74 years receiving radiotherapy for MM of the head and neck were included in the retrospective analysis. 19 patients were treated with bimodal radiotherapy, 3 patients with photon only radiotherapy. Before initiation of radiotherapy, staging was conducted using the eighth edition of the Union for International Cancer Control (UICC) TNM system. Most frequent sites of primary tumor were multiple paranasal sinus (9 patients, 41%), nasal cavity (6 patients, 27%) and maxillary sinus (2 patients, 9%). Detailed patient characteristics are described in **Table 1**.

**Table 1:** Patient characteristics.

Parameter	No. (%)
Median age	74 (48–90) years
Sex	
Male	7 (32)
Female	15 (68)
Primary tumor site	
Nasal cavity	6 (27)
Multiple paranasal sinus	9 (41)
Maxillary sinus	2 (9)
Buccal mucosa	1 (5)
Ethmoid sinus	1 (5)
Hard palate	1 (5)
Nasopharynx	1 (5)
Sphenoidal sinus	1 (5)

### 2.3 Treatment features

The majority of patients received surgical treatment before radiotherapy (14 patients, 64%). For most patients who underwent surgery, treatment planning revealed substantial remaining disease classified as R2 (7 patients, 50%). However, R1 resections were achieved in 6 cases (43%). One patient did not show any microscopic residual disease (R0, 7%).

For radiotherapy, immobilization of patients was done using a thermoplastic head-mask system with shoulder fixation. Treatment planning was based on computed tomography (CT) scans with 1.0 mm slice thickness and contrast-enhanced magnetic resonance imaging (MRI). Target volumes were defined according to standard procedures at our institution. We delineated two target volumes as follows: Clinical Target Volume (CTV)1 encompassed the macroscopic tumor along with the tumor bed with a 5-7 mm margin adjusted to anatomical boundaries. In addition to CTV1, CTV2 incorporated the typical pathways of spread and included elective nodal levels following the guidelines of Biau et al. depending on primary site, tumor size and nodal status (10). In cases where the primary tumor extended across or was located at the midline, bilateral nodes were encompassed. For the creation of planning target volumes (PTVs), we applied a 3-mm margin around the CTVs. However, in situations where extending this margin would encroach upon critical structures (such as the optic system), we either reduced or omitted the margin accordingly.

C12 treatment plans were generated using Siemens syngo RT Planning, which utilizes a biological plan optimization approach based on the local effect model to consider enhanced biological effectiveness equivalent to Gy(RBE, radiobiological effectiveness) (11, 12). This model doesn’t account for variations in biological effectiveness caused by hypofractionation. To obtain the biological equivalent dose in Gy(RBE), doses must be converted following the standard linear quadratic (LQ) model. Photon treatment plans were generated using MRC Kon-Rad on the Siemens Syngo platform, either for step-and-shoot intensity-modulated radiotherapy (IMRT) or tomotherapy for helical IMRT.

Bimodal radiotherapy consisted of IMRT of the primary tumor region and cervical lymph nodes (CTV2/PTV2) in a single dose of 2 Gy up to the dose of 50.0 Gy in nodal negative patients or 56 Gy in nodal positive patients in the basic schedule after application of a C12 boost of the primary tumor region (CTV1/PTV1) up to 24 Gy(RBE) in nodal negative patients or up to 18 Gy (RBE) in nodal positive patients in a single dose of 3 Gy(RBE) up to the total dose of 74.0 Gy(RBE) in 5-6 fractions/week. The doses for both C12 and photons were prescribed to the median PTV, ensuring that 95% of the target volume were encompassed by the prescription isodose.

Moreover 3 patients were treated with photon only IMRT since insurance did not cover particle therapy expenses. IMRT up to total doses of 66-70,4 Gy in 5 fractions/week was used in single doses of 2-2.2 Gy.

Regarding systemic therapy, 3 patients (18%) received adjuvant pembrolizumab 200 mg fix dose for one year and one patient (5%) underwent adjuvant ipilimumab/nivolumab after completion of radiotherapy. Only one patient (5%) received neoadjuvant treatment with 4 cycles of carboplatin/paclitaxel before start of irradiation.

### 2.4 Follow-up

Patients’ follow-up examinations were scheduled every three months during the first two years after the end of radiotherapy, every six months during the following two years and then once a year with contrast-enhanced MRI or CT scans of the head and neck. CT scans of the chest and ultrasound of the abdomen were performed annually. A radiation oncologist recorded current symptoms and toxicities related to treatment at each follow-up visit. Clinical examination by an ear, nose and throat specialist was performed regularly. Evaluation of toxicities related to treatment was done from medical records using the Common Terminology Criteria for Adverse Events (CTCAE) version 5.

Calculation of time-to-event data (OS, PFS, LCR and LRCR) was done from the first date of histopathological diagnosis to the date of the last follow-up, death or time of event (local, regional or distant progress) using the Kaplan-Meier method (IBM SPSS statistics version 27). Local treatment failure was defined as recurrence at the primary tumor site. Events for LRCR were recurrence at the primary tumor site and/or regional failure as recurrence in cervical lymph nodes.

## 3 Results

### 3.1 Treatment outcome

For all patients, median follow-up was 18 months (range 7 – 117 months).

**Figure 1:**
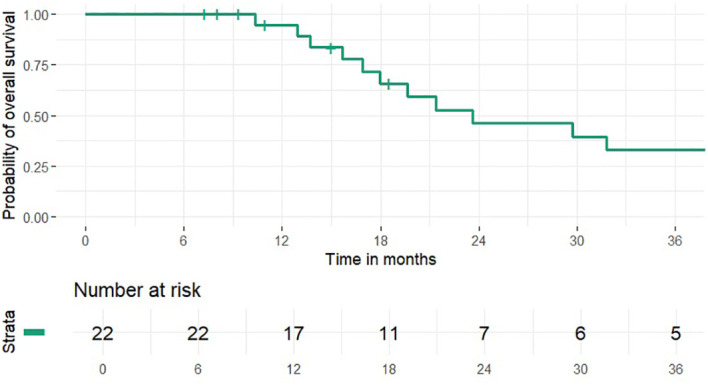
Overall survival.

**Figure 2:**
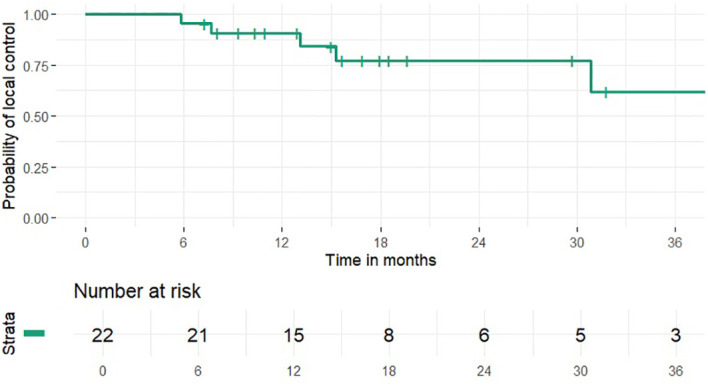
Local control rate.

**Figure 3:**
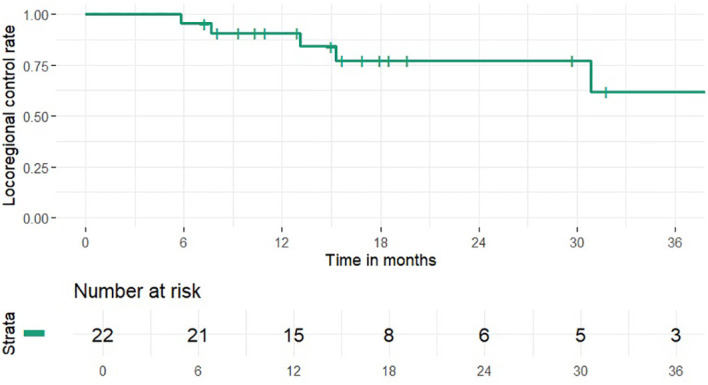
Locoregional control rate.

**Figure 4:**
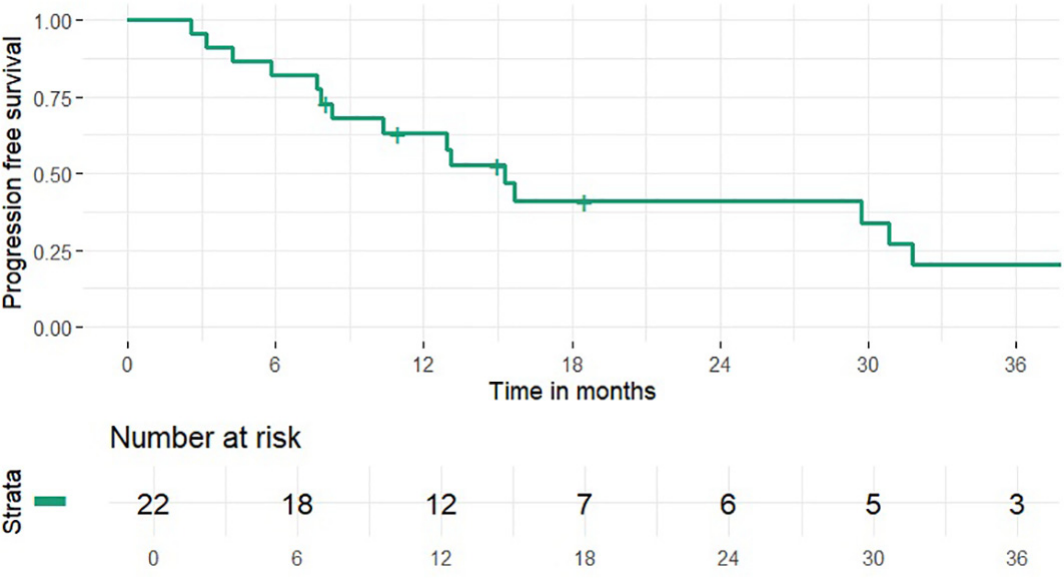
Progression free survival.

1 and 2-year OS rates were 95% and 46% (median 24 months), PFS rates were 63% and 41% (median 15 months), 1 and 2-year LCR rates were 91% and 77%, LRCR rates were 91% and 77%, respectively (**Figures 1**-**4**). At the time of their last follow-up examination, 13 patients (59%) were dead. Local, regional or distant failure after treatment was reported in 10 patients (45%). Treatment failure manifested always within the first three years after treatment.

**Figure 5:**
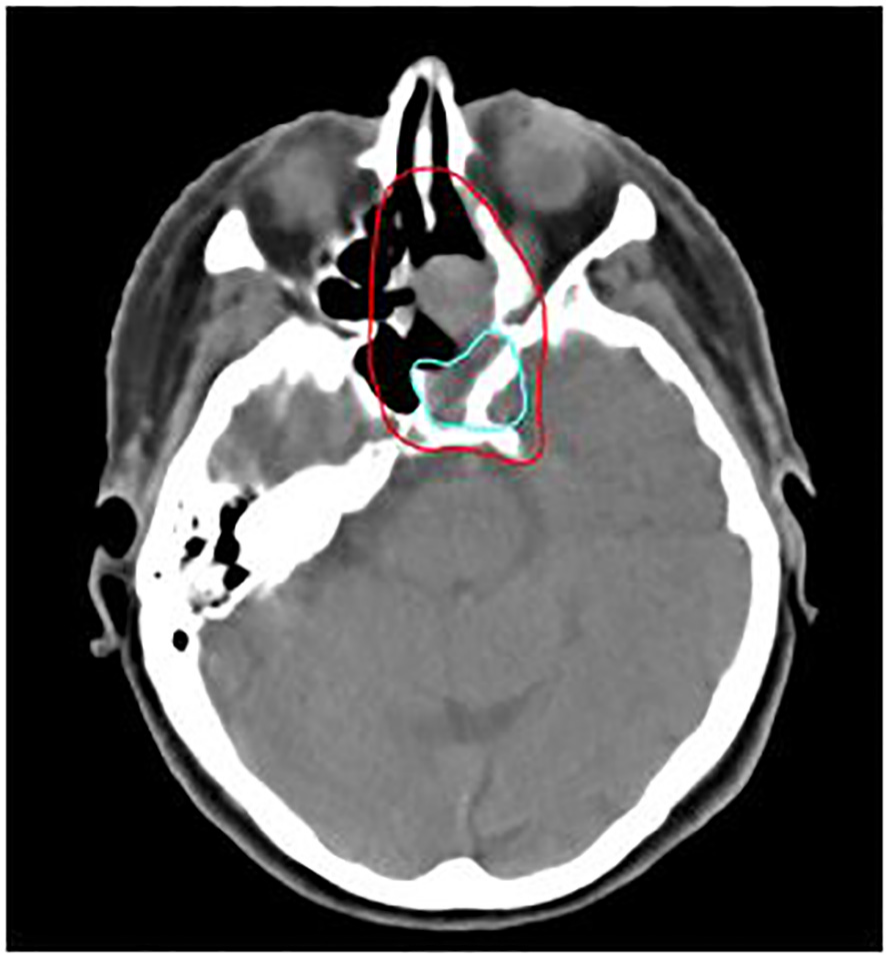
76-year-old female patient with mucosal melanoma of the paranasal sinus treated with bimodal intensity-modulated radiotherapy (IMRT) in combination with carbon ion therapy. The patient developed a local relapse (light blue) located in-field of the C12 boost CTV (red), delineated on the planning CT.

The major failure pattern after treatment was distant metastasis (8 cases, 36%). Most frequent sites of metastasis were pulmonary (6 cases), osseous (5 cases), hepatic (4 cases) and adrenal (2 cases). Local recurrence was the secondary pattern of failure (5 cases, 23%). 4 out of 5 local relapses were in-field of C12 boost volume. A clinical case example of a patient with an local relapse located in-field of C12 boost volume is shown in **Figure 5**. The primary tumor of all of the 4 patients with local in-field relapses was located in the main nasal cavity and/or in the paranasal sinus. Moreover, 1 patient (5%) showed distant metastasis as well as local and regional recurrence.

Within the 3 patients treated with photon only IMRT, 2 patients showed treatment failure: 1 patient showed distant progression, the other one showed local progression.

### 3.2 Acute toxicity

Acute toxicities comprised adverse events that occurred from the start of radiation until 90 days after. Toxicities were evaluated according to the Common Terminology Criteria of Adverse Events (CTCAE). All patients completed bimodal radiotherapy without interruptions. Bimodal radiotherapy showed high tolerability without adverse events higher than grade 3. Grade 3 acute toxicity was found in 9 patients (41%) with mucositis with severe pain and interference with oral intake being the most reported grade 3 adverse event (3 patients, 14%). The most common acute toxicities were grade 1 radiation dermatitis (10 patients, 45%) and grade 2 mucositis (12 patients, 55%).

### 3.3 Late toxicity

Late toxicity included adverse events reported more than 90 days after initiation of chemoradiotherapy. No patient presented grade 4 or 5 late toxicity. Most of the described grade 3 acute adverse events had resolved until the first or second follow-up examination after treatment. 2 patients (15%) suffered from grade 3 central nervous system necrosis. Medical intervention with usage of dexamethasone/bevacizumab was necessary and resulted in full recovery. Moreover, one patient developed an early-onset secondary malignancy, possibly radiation induced. The patient was diagnosed with a pathologically confirmed osteosarcoma in the former boost volume 2.5 years after bimodal radiotherapy of a mucosal melanoma of the nasal cavity. Therapy following the best supportive care approach was initiated, and the patient passed away after an additional four months. The most common late adverse events comprised grade 1 xerostomia (5 patients, 38%) and grad 1 dysgeusia and mucositis (4 patients each, 23%). Acute and late toxicity in detail is shown in **Table 2**.

**Table 2:** Toxicity.

Toxicity	CTCAE Grade		
	1	2	3
Acute			
Xerostomia	6 (27)	6 (27)	0
Dysgeusia	4 (18)	6 (27)	0
Dysphagia	2 (9)	6 (27)	2 (9)
Mucositis	5 (23)	12 (55)	3 (14)
Nausea	3 (14)	0	0
Lymphedema	1 (5)	0	0
Dermatitis radiation	10 (45)	3 (14)	2 (9)
Tympanic effusion Fatigue	0 3 (14)	1 (5) 2 (9)	0 1 (5)
Conjunctivitis	3 (14)	5 (23)	0
Optic nerve disorder	1 (5)	0	2 (9)
Late			
Xerostomia	5 (38)	0	0
Dysgeusia	3 (23)	0	0
Mucositis	3 (23)	1 (8)	0
Hyposmia	2 (15)	0	0
Synechia	0	2 (15)	0
Tympanic effusion	1 (8)	0	0
Fatigue	1 (8)	0	0
CNS necrosis	0	0	2 (15)
Secondary malignancy	0	0	1 (8)

## 4 Discussion

The management of mucosal melanoma in the head and neck region continues to present unique challenges. Current guidelines recommend surgical resection as the primary treatment for mucosal melanoma when feasible. However, guidelines also indicate postoperative radiotherapy as part of treatment in certain scenarios e.g. when extranodal extension, involvement of at least two lymphatic nodes, any node 3 cm or greater, or when relapse after primary surgical resection is present (13). When choosing radiotherapy technique, one has to consider the relative radio resistance of mucosal melanoma, making local control dose-dependent (14). Particle therapy provides precise dose distributions and the possibility of increasing the dosage. Neutrons and C12 also offer enhanced biological effectiveness (15, 16). Moreover, they are less influenced by the oxygen effect, making them advantageous for treating large and hypoxic tumors.

In this study the combination of IMRT with carbon ion therapy in the treatment of mucosal melanoma lead to an OS rate of 46%, a LCR rate of 77%, LRCR rate of 77% and a PFS rate of 41% at 2 years, respectively. Data from our clinic from 2015 showed 32.3%, 39.1% and 77.7% for OS, PFS and LRCR after the same time (17). Thus, within the clinic treatment OS and PFS seemed to be similar to gradually better and LRCR did not change compared to the results 10 years ago. Further research could try to identify possible causes for the slight differences in outcome e.g. differences in the radiotherapeutic or systemic therapy approach.

The ability to control the tumor locally in this study is impressive compared to other experiences with particle therapy, despite the fact that most of the patients have advanced tumor stages and substantial residual disease. It is worth noting that there was no case of cervical nodal metastases after radiotherapy using the bimodal treatment approach, unlike other institutions where rates of nodal failures were reported to be higher (17.7%, 18.1%, and 28.6%) (9, 18, 19). This supports our decision to include regional nodal levels in the expanded target volume for treatment.

An interesting finding of this study is the location of the primary tumor when local in-field relapse occurred: All instances of local in-field recurrence within the C12 boost volume were confined to patients with primary neoplasms localized within the primary nasal cavity or the paranasal sinus. This observation prompts speculation regarding the influence of tumor depth on treatment response, particularly in the context of particle beam therapy. The diminished efficacy observed in superficially situated tumors may be attributable to the absence of a discernible dose build-up effect inherent to particle therapy. Consequently, superficial tumors may be more susceptible to treatment failure and subsequent recurrence due to underdosage.

An increasing body of research indicate the noteworthy advantages of utilizing radiotherapy as a complementary strategy to enhance the effectiveness of immunotherapy in treating cutaneous melanoma. At the same time, concomitant immunotherapy during radiotherapy is often feared by physicians because of possible aggravation of toxicity. Thus, different research groups tried to investigate combination of immunotherapy such as anti-PD1 or anti-CTLA4 antibodies with irradiation in mucosal melanoma (20, 21). E.g. median PFS was twice as long (8.9 vs 4.2 months) for patients with mucosal melanoma treated with the combination of anti-PD1 antibodies and radiotherapy compared to immunotherapy alone (20). However, current literature lacks of high number of investigated patients and is mostly of retrospective nature. Moreover, reports about toxicity of combining immunotherapy and irradiation in mucosal melanoma is scarce. Results of ongoing trials (e.g. NCT04017897) are highly anticipated. In contrast, this study does not comprise any patients with concomitant irradiation and immunotherapy due to the difficulty to predict treatment toxicity. As soon as more data on this topic is available this might become a new topic of discussion in multidisciplinary team meetings.

Regarding acute toxicity, bimodal treatment was tolerable in our patient cohort. There was no acute toxicity higher than grade III (**Table 2**). With 14% of patients suffering from grade III mucositis, acute toxicity in this study is similar to previous literature on this matter (17, 19, 22). In general, most of acute toxicities had been cured in first or second follow-up examination in our study. Nonetheless, relevant late toxicities like e.g. grade III CNS necrosis were observed in 2 patients. Fortunately, treatment of CNS necrosis lead to full recovery of the afflicted patients. However, this observation underlines the need for thorough follow-up examinations after irradiation of patients with mucosal melanoma of the head and neck. Late toxicity of grade III or higher was observed in 5% to 14% of patients in other studies (18, 19, 22). Hence, our results seem to be in line with previously published literature. The fact, that target volumes in the patient cohort of our study might have been larger compared to other institutions due to the inclusion of cervical lymph nodes, should be considered when comparing toxicity results to other reports.

When interpreting the results of this study, several limitations must be taken into account. The current study is of retrospective nature and did not compare the results using a prospective randomized trial. However, prospective randomized trials in patients with mucosal melanoma of the head and neck are scarce. Multicenter studies are needed. Despite of the long follow-up time frame of up to 117 months at the most, 3 of the initial 22 patients (14%) were lost to follow-up. Nevertheless, the presented data seems to be interesting considering the fact that there is already published data from our institution reporting about success rates and tolerability of IMRT combined with carbon ion therapy (17). Moreover, possible combination of bimodal radiotherapy with immunotherapy was not tested at our clinic so far. Further studies could focus especially on the investigation of treatment outcomes and evaluation of possible adverse events of radioimmunotherapy. Furthermore, the rarity of mucosal melanomas and the lack of comprehensive studies regarding this disease may lead to the recommendation to treat all afflicted patients in prospective trials in order to facilitate therapy optimization.

## Data Availability

The raw data supporting the conclusions of this article will be made available by the authors, without undue reservation.

## References

[1] McLaughlin CC Wu X Jemal A Martin HJ Roche LM Chen VW. Incidence of noncutaneous melanomas in the U.S. Cancer. (2005) 103:1000–7. doi: 10.1002/cncr.v103:5 15651058

[2] Bishop KD Olszewski AJ. Epidemiology and survival outcomes of ocular and mucosal melanomas: A population-based analysis. Int J Cancer. (2014) 134:2961–71. doi: 10.1002/ijc.v134.12 24272143

[3] Wolchok JD Chiarion-Sileni V Gonzalez R Grob JJ Rutkowski P Lao CD. Long-term outcomes with nivolumab plus ipilimumab or nivolumab alone versus ipilimumab in patients with advanced melanoma. J Clin Oncol. (2022) 40:127–37. doi: 10.1200/JCO.21.02229 PMC8718224 34818112

[4] Ho J Mattei J Tetzlaff M Williams MD Davies MA Diab A. Neoadjuvant checkpoint inhibitor immunotherapy for resectable mucosal melanoma. Front Oncol. (2022) 12:1001150. doi: 10.3389/fonc.2022.1001150 36324592 PMC9618687

[5] Jensen AD Nikoghosyan AV Ecker S Ellerbrock M Debus J Münter MW. Carbon ion therapy for advanced sinonasal Malignancies: feasibility and acute toxicity. Radiat Oncol Dezember. (2011) 6:30. doi: 10.1186/1748-717X-6-30 PMC3080287 21466696

[6] Amirul Islam M Yanagi T Mizoe J Mizuno H Tsujii H. Comparative study of dose distribution between carbon ion radiotherapy and photon radiotherapy for head and neck tumor. Radiat Med August. (2008) 26:415–21. doi: 10.1007/s11604-008-0252-9 18769999

[7] Wang X Chen X Li G Han X Gao T Liu W. Application of carbon ion and its sensitizing agent in cancer therapy: A systematic review. Front Oncol. (2021) 11:708724. doi: 10.3389/fonc.2021.708724 34290989 PMC8287631

[8] Takayasu Y Kubo N Shino M Nikkuni O Ida S Musha A. Carbon-ion radiotherapy combined with chemotherapy for head and neck mucosal melanoma: Prospective observational study. Cancer Med. (2019) 8:7227–35. doi: 10.1002/cam4.v8.17 PMC6885871 31621203

[9] Yanagi T Mizoe J Hasegawa A Takagi R Bessho H Onda T. Mucosal Malignant melanoma of the head and neck treated by carbon ion radiotherapy. Int J Radiat Oncol. (2009) 74:15–20. doi: 10.1016/j.ijrobp.2008.07.056 19046826

[10] Biau J Lapeyre M Troussier I Budach W Giralt J Grau C. Selection of lymph node target volumes for definitive head and neck radiation therapy: a 2019 Update. Radiother Oncol. (2019) 134:1–9. doi: 10.1016/j.radonc.2019.01.018 31005201

[11] Krämer M Scholz M. Treatment planning for heavy-ion radiotherapy: calculation and optimization of biologically effective dose. Phys Med Biol. (2000) 145:3319–30. doi: 10.1088/0031-9155/45/11/314 11098906

[12] Elsässer T Krämer M Scholz M. Accuracy of the local effect model for the prediction of biologic effects of carbon ion beams in vitro and *in vivo* . Int J Radiat Oncol. (2008) 71:866–72. doi: 10.1016/j.ijrobp.2008.02.037 18430521

[13] Pittaka M Kardamakis D Spyropoulou D. Comparison of international guidelines on mucosal melanoma of the head and neck: A comprehensive review of the role of radiation therapy. Vivo Athens Greece. (2016) 30:165–70. 27107071

[14] Moreno MA Roberts DB Kupferman ME DeMonte F El-Naggar AK Williams M. Mucosal melanoma of the nose and paranasal sinuses, a contemporary experience from the M. D. Anderson Cancer Center. Cancer. (2010) 116:2215–23. doi: 10.1002/cncr.v116:9 20198705

[15] Ferrari M Orlandi E Bossi P. Sinonasal cancers treatments: state of the art. Curr Opin Oncol. (2021) 33:196–205. doi: 10.1097/CCO.0000000000000726 33756515 PMC9904434

[16] Franz L Zanoletti E Nicolai P Ferrari M. Treatment of skull base diseases: A multidisciplinary challenge. J Clin Med. (2023) 12:1492. doi: 10.3390/jcm12041492 36836028 PMC9966366

[17] Mohr A Chaudhri N Hassel JC Federspil PA Vanoni V Debus J. Raster-scanned intensity-controlled carbon ion therapy for mucosal melanoma of the paranasal sinus. Head Neck. (2016) 38:E1445–51. doi: 10.1002/hed.24256 26560744

[18] Demizu Y Fujii O Terashima K Mima M Hashimoto N Niwa Y. Particle therapy for mucosal melanoma of the head and neck: A single-institution retrospective comparison of proton and carbon ion therapy. Strahlenther Onkol. (2014) 190:186–91. doi: 10.1007/s00066-013-0489-9 24362502

[19] Liao JJ Parvathaneni U Laramore GE Thompson JA Bhatia S Futran ND. Fast neutron radiotherapy for primary mucosal melanomas of the head and neck. Head Neck. (2014) 36:1162–7. doi: 10.1002/hed.23428 23852725

[20] Teterycz P Czarnecka AM Indini A Spałek MJ Labianca A Rogala P. Multimodal treatment of advanced mucosal melanoma in the era of modern immunotherapy. Cancers. (2020) 12:3131. doi: 10.3390/cancers12113131 33114734 PMC7692305

[21] Smart AC Giobbie-Hurder A Desai V Xing JL Lukens JN Taunk NK. Multicenter evaluation of radiation and immune checkpoint inhibitor therapy in mucosal melanoma and review of recent literature. Adv Radiat Oncol. (2024) 9:101310. doi: 10.1016/j.adro.2023.101310 38260223 PMC10801653

[22] Fuji H Yoshikawa S Kasami M Murayama S Onitsuka T Kashiwagi H. High-dose proton beam therapy for sinonasal mucosal Malignant melanoma. Radiat Oncol. (2014) 9:162. doi: 10.1186/1748-717X-9-162 25056641 PMC4118609

